# Nutrigenomic analyses reveal miRNAs and mRNAs affected by feed restriction in the mammary gland of midlactation dairy cows

**DOI:** 10.1371/journal.pone.0248680

**Published:** 2021-04-15

**Authors:** Pierre-Alexis Billa, Yannick Faulconnier, Tao Ye, Céline Bourdon, José A. A. Pires, Christine Leroux

**Affiliations:** 1 INRAE, Université Clermont Auvergne, VetAgro Sup, UMR Herbivores, Saint-Genès-Champanelle, France; 2 Institut de Génétique et de Biologie Moléculaire et Cellulaire (IGBMC), Centre National de la Recherche Scientifique, UMR7104, Institut National de la Santé et de la Recherche Médicale, U964, Université de Strasbourg, Illkirch, France; 3 INRAE, AgroParisTech, Université Paris-Saclay, UMR Génétique Animale et Biologie Intégrative, Jouy-en-Josas, France; Michigan State University, UNITED STATES

## Abstract

The objective of this study was to investigate the effects of feed restriction on mammary miRNAs and coding gene expression in midlactation cows. Five Holstein cows and 6 Montbéliarde cows underwent 6 days of feed restriction, during which feed allowance was reduced to meet 50% of their net energy for lactation requirements. Mammary biopsies were performed before and at the end of the restriction period. Mammary miRNA and mRNA analyses were performed using high-throughput sequencing and microarray analyses, respectively. Feed restriction induced a negative energy balance and decreased milk production and fat and protein yields in both breeds. Feed restriction modified the expression of 27 miRNAs and 374 mRNAs in mammary glands from Holstein cows, whereas no significant miRNA change was observed in Montbéliarde cows. Among the 27 differentially expressed miRNAs, 8 miRNAs were associated with dairy QTL. Analysis of target genes indicate that the 8 most abundantly expressed miRNAs control transcripts related to lipid metabolism, mammary remodeling and stress response. A comparison between the mRNAs targeted by the 8 most strongly expressed miRNAs and 374 differentially expressed mRNAs identified 59 mRNAs in common. The bioinformatic analyses of these 59 mRNAs revealed their implication in lipid metabolism and endothelial cell proliferation. These effects of feed restriction on mammary miRNAs and mRNAs observed in Holstein cows suggest a potential role of miRNAs in mammary structure and lipid biosynthesis that could explain changes in milk production and composition.

## Introduction

The mammary gland (**MG**) is a complex secretory organ with the ability to synthesize and secrete large quantities of milk components, including proteins, lactose and lipids. Genetics, nutritional and physiological studies of MG functioning have shown that many genes regulate the synthesis and secretion of milk components [1]. Forty-eight hours of feed deprivation induced a severe negative energy balance (**NEB**) and altered the mammary transcriptome, milk production and composition in lactating goats [2]. However, the molecular mechanisms underlying the nutritional regulation of gene expression have not been fully determined.

MicroRNAs (**miRNAs**), small noncoding RNAs of 18–25 nucleotides, regulate at least 60% of gene expression by base-pairing with mRNAs, inducing their degradation or the inhibition of their translation [3]. Therefore, the RNAs are involved in many different cellular processes [4–6]. MiRNAs were studied in the MG of different species, including ruminants [7–12], and their potential role in mammary development [8] and milk synthesis [13] was demonstrated. Breed effects on miRNA expression were also studied in different tissues, including MG [14–16]. In a previous study, the comparison of mammary miRNomes from Holstein (**HOLS**) and Montbéliarde (**MONT**) cows revealed 22 differentially expressed miRNAs (**DEMs**), suggesting a potential role of miRNAs in MG plasticity and milk component synthesis [15].

In ruminants, few studies have investigated the effect of nutrition on miRNA expression in the MG. Feed deprivation leading to severe NEB modified the expression of 30 miRNAs in MG of lactating goats [10].

We hypothesized that bovine mammary miRNome would be modified by feed restriction. Thus, the objective was to determine the effects of NEB induced by feed restriction on MG miRNome, assess potential differences between midlactation HOLS and MONT cows, and determine the role of miRNAs in the regulation of milk production and milk component synthesis.

## Materials and methods

### Ethics statement

The experiment was conducted at the INRAE Herbipole experimental farm of Marcenat (https://doi.org/10.15454/1.5572318050509348E12). Animal procedures were performed in compliance with Regional Animal Care Committee guidelines CEMEAA: Auvergne, French Ministry of Agriculture and European Union guidelines for animal research C2EA-02. All mammary biopsies were performed with relevant national legislation and were performed by an accredited person (N° of certification: 63–20). All procedures involving animals were approved by the Ethics Committee of the French Ministry of Higher Education, Research and Innovation (APAFIS # 3737–2015043014541577v2).

### Animals and sampling

Twelve multiparous midlactation HOLS (n = 6; 2.5 ± 0.8 lactations and 170 ± 11 days in milk (**DIM**)) and MONT (n = 6; 3.3±2.0 lactations and 156 ± 26 DIM) cows were used to study the effects of 6 days of feed restriction on milk production and mammary miRNomes. One HOLS cow was excluded from the experiment due to mastitis. During the control period (**CONT**) before restriction, all cows were allowed *ad libitum* intake of a total mixed ration. During the restriction period (d1 to 6, **REST**), feed allowance was reduced to meet 50% of NE_L_ requirements calculated from body weight, feed intake and milk production and composition recorded before restriction [17]. Cows had free access to water and were housed in a freestall barn equipped with automatic feed bunks that control individual access and weight feed intake (CRFI, Biocontrol, Rakkestad, Norway). Cows had free access to water and were milked twice daily at approximately 6:30 and 16:00. The nutrient composition of the experimental diets was previously described [18].

MG samples were collected by biopsies for miRNome analyses on day 0 and day 6 relative to initiation of feed restriction. Mammary biopsies were performed as previously described [19]. Briefly, the biopsy site was selected at a midpoint on alternate rear quarters of mammary gland. Approximately 600–650 mg of tissue was collected, rinsed in sterile 0.9% saline solution, snap-frozen in liquid nitrogen, and stored at −80°C until RNA extraction.

Milk samples were collected on day -1 and on day 5 relative to initiation of feed restriction. Milk composition was determined by mid-infrared spectroscopy (LIAL, Aurillac, France), and their means were computed according to PM/AM production and composition. Milk production and energy balance data were analyzed using mixed models of SAS (version 9.4; SAS Institute Inc., Cary, NC) that included day, breed, and their interaction as fixed effects, cow as random effect, and Kenward-Roger adjustment for calculation of degrees of freedom.

### RNA preparation

Total RNA was extracted from approximately 50 mg of MG biopsies using the miRVana kit (Thermo Fisher Scientific, USA). The concentration and quality of the RNAs were estimated by spectrophotometry Nanodrop TH (ND-1000, NanoDrop Technologies LLC, Wilmington, DE, USA) and using the Bioanalyzer 2100 (Agilent Technologies Inc., Santa Clara, CA, USA), respectively. The means of the obtained RIN were 8.3 ± 0.6 and 8.3 ± 0.9 in HOLS and MONT, respectively.

### RNA-seq analysis

RNA-seq analyses were performed on RNAs from MG of five HOLS and six MONT cows before and at the end of the restriction period, by the IGBMC Microarray and Sequencing Platform (Strasbourg, France), as previously described [15]. Briefly, small RNA-seq libraries were generated from 2 μg of total RNA using the TruSeq Small RNA Library Prep Kit (Illumina, San Diego, CA) according to the manufacturer’s instructions. Specific miRNA and small RNA adapters were added to each end of the miRNA followed by reverse transcription and PCR amplification to obtain cDNA. Acrylamide gel purification of small RNAs was performed. The final cDNA libraries were sequenced on HiSeq 4000 (Illumina) as single-end 50 base reads according to the manufacturer’s instructions. Adaptor sequences and reads with undetected bases were removed (FASTX-Toolkit, http://hannonlab.cshl.edu/fastx_toolkit/index.html), and all remaining reads were filtered according to their size. Read quantification and annotation were performed using the ncPRO-seq pipeline [20]. The sequence reads were aligned against the *Bos taurus* btau5.0.1 genome as miRBase_v22.1 using the miRDeep2 package [21]. Precursors and mature miRNAs were identified using the miRDeep2 core module, miRDeep2.pl. Potential miRNAs were annotated accordingly against orthologous miRNAs in goat, sheep and humans (miRBase release 22.1). We used a miRDeep2 score ≥ 0 as a cut-off threshold. The normalization and differential expression analyses of miRNAs were conducted using the DESeq2 R package v1.18.1. Significance was considered at P_adj_ ≤ 0.05. The accession number of the RNA-seq data is GSE146762.

### Microarray analyses

Due to the miRNA results, mRNA analyses were performed only in HOLS. Fifty nanograms of total RNA from the MG samples (n = 12) already used for miRNA analyses were analyzed using a three bovine 4 × 44K microarray (Agilent Technologies, Inc., Santa Clara, CA, USA) leading to 12 arrays. All procedures were performed according to the manufacturer’s instructions. Briefly, total RNA was linearly amplified and labeled with Cy3 using the one-color Low Input Quick Amp Labeling Kit (Agilent Technologies, Inc. Santa Clara, CA, USA). Cy3-labeled cRNA (1650 ng) was hybridized using the Gene Expression Hyb Kit (Agilent Technologies, Inc., Santa Clara, CA, USA). Hybridization was performed for 17 h at 65°C in a rotating hybridization oven at 10 rpm. Following hybridization, all microarrays were washed and scanned using the Agilent Microarray Scanner G2565A (Agilent Technologies, Inc. Santa Clara, CA, USA). The resulting TIFF images (Tagged Image File Format) were processed using Feature Extraction software Version 11. Microarrays were analyzed to determine differential gene expression (**DEG**) at the mRNA level using GeneSpring software, and data were normalized with 75^th^ percentile shift followed by *t*-tests moderated with the Benjamini-Hochberg (FDR) correction [22], and significance was considered at P_adj_ < 0.10. Microarray data were deposited in the Gene Expression Omnibus (GEO): GSE159302.

### Bioinformatic analyses

The genomic localization of DEMs was compared with the genomic location of milk-associated QTL. All bovine QTL downloaded from CattleQTLdb (AnimalQTLdb release 40, December 29, 2019, https://www.animalgenome.org) were obtained from the "All data by bp (on UMD3.1)" link in bed format. These localizations were used to identify the mammary miRNAs hosted in these regions. The genomic positions of the miRNAs in the UMD3.1 reference genome permitted their screening in the QTL regions using a homemade bioinformatics script in Python language (S1 Fig). The QTL related to milk production and composition were then extracted.

Target genes of **DEMs** and corresponding putative biological processes (P ≤ 0.05) were investigated using the miRWalk database (version 3.0) and Enrichr platform [23, 24], integrating several tools for pathway visualizations, including KEGG, BioCarta, for more efficient analyses of genes in networks. Modulated biological processes were grouped using the REVIGO platform (http://revigo.irb.hr/) [25]. Metacore^TM^ software (release 6), which uses text mining algorithms to identify interactions and build pathways/networks, was also used to corroborate bioinformatics investigations. However, these algorithms do not use the exact representativeness corresponding to the background of the used whole genome microarray. A Venn diagram was obtained using Venny 2.1 (http://bioinfogp.cnb.csic.es/tools/venny/) to find common genes between miRNA target genes and DEGs identified by microarray analyses. Biological processes (P ≤ 0.05) of these common genes were investigated using the Enrichr platform for both up and down-regulated genes together.

## Results and discussion

### Animal responses and milk analyses

Energy balance, milk production and composition were analyzed to characterize cow response to the feed restriction model (Table 1). Feed restriction induced NEB in both HOLS and MONT cows (-35 and -29 ± 4 MJ/day, respectively) due to the experimental design. Milk production, fat and protein yields decreased by 40, 33 and 43% in HOLS and 43, 34 and 49% in MONT, respectively, during REST (Table 1). Production and metabolic responses are detailed elsewhere [18]. The effects of restriction on milk production are in agreement with previous studies in mid- and late-lactation HOLS cows [26, 27].

**Table 1:** Effects of feed restriction on energy balance, DMI, milk, milk fat and protein yields in midlactation Holstein (HOLS) and Montbéliarde (MONT) cows during control (CONT) and restriction (REST) periods.

	Breed	Period		SEM	P-value		
		CONT	REST		Breed	Period	Breed x Period
Energy balance (MJ/d)	HOLS	56.5^a^	-35.1	4.2	0.03	<0.001	<0.001
	MONT	26.0^b^	-28.9	3.9			
Milk yield (kg/d)	HOLS	30.8	18.5	1.0	<0.001	<0.001	0.38
	MONT	25.1	14.2	0.9			
Fat yield (g/d)	HOLS	1 030.1	694.4	32.1	0.005	<0.001	0.62
	MONT	896.2	590.3	29.3			
Protein yield (g/d)	HOLS	929.3	529.1	33.9	0.008	<0.001	0.82
	MONT	795.6	407.4	30.9			
Lactose yield (g/d)	HOLS	1 565.3	970.7	50.8	<0.001	<0.001	0.96
	MONT	1 263.6	665.8	44.3			

SEM corresponds to the standard error of the mean. a, b correspond to breed LSMEANS not sharing a common superscript differ between CONT and REST periods (*P* ≤ 0.05) and are presented when Breed × Period effect was significant.

A significant breed effect was observed for energy balance and production variables. Montbéliarde cows had lower milk, fat and protein yields than HOLS cows, which is in agreement with previous studies [28, 29]. A significant period by breed interaction was observed for energy balance and because energy balance was lower in MONT during the CONT period compared to HOLS cows. This could be due to lower intake in MONT than in HOLS cows during this period [18].

### Mammary gland miRNome responses

#### Summary of miRNA sequencing data

Ten and twelve libraries were constructed using RNA extracted from the MG of HOLS and MONT cows at CONT and at REST periods. More than 12 million raw reads and more than 8.5 million clean reads were obtained after removing adapters, no-clipped reads and reads that were too short. The percentage of clean reads mapped into the *Bos taurus* genome was 97.4 on average for all libraries. In HOLS and MONT cows, the log_2_ of the normalized counts of the CONT and REST libraries had a strong correlation of R ≥ 0.96. MiRdeep2 software analysis identified 754 miRNAs (737 known and 17 predicted) for HOLS libraries and 759 miRNAs (743 known and 16 predicted) for MONT libraries. These results are in accordance with other studies on bovine MG using RNA-seq technology, reporting 654 [9] and 324 [11] known miRNAs. However, these studies presented a higher number of predicted miRNAs (679 in Le Guillou et al. [9] and 176 in Li et al. [11]) than in our study. The difference could be due to the version of miRBase used (miRBase V20, V21 and V22 in Le Guillou et al. [9], Li et al. [11] and our study, respectively). In the present study, the output ratio of CONT compared to the REST period was closer in HOLST than in MONT libraries (Δ1.6% and Δ6.4%, respectively; Table 2).

**Table 2:** Effect of feed restriction on miRNA RNA-seq data in mammary glands from midlactation Holstein (n = 5) and Montbéliarde (n = 6) cows in control (CONT) and restriction (REST) periods.

Breed	Condition	Raw read	Too short	Adapter	Clean read	Output ratio (%)
Holstein	CONT	13,393,626	724,538	56,192	11,027,924	82.2
	REST	17,101,298	794,008	61,674	14,352,279	83.8
Montbéliarde	CONT	11,953,902	1,405,670	824,394	8,500,865	71.0
	REST	14,841,154	1,354,192	622,388	11,539,527	77.4

Clean reads were obtained after size (18 to 25 nt), adapter and soft-clipping cleaning.

#### Differentially expressed miRNAs between the control and restriction periods

The expression of 27 miRNAs was significantly modified by feed restriction in HOLS cows (P_adj_ ≤ 0.05; Table 3), but no significant miRNA change was observed in MONT cows. The absence of DEMs in MONT cows may be due to the lower quality of their libraries compared to those of HOLS cows (with a greater number of short reads and adapters and thus a smaller output ratio in MONT; Table 3). Another hypothesis is that MONT cows presented a lower energy value during the CONT period than HOLS cows. Montbéliarde cows presented a lower variation of energy balance between the CONT and REST periods (26 MJ in CONT and -28.9 MJ in REST: Δ54.6 MJ) compared to HOLS cows (56.5 MJ in CONT and –35.1 MJ in REST: Δ91.6 MJ). In addition, MONT cows had a lower decrease in the milk, fat, and protein yields than HOLS. Moreover, decades of genetic selection in HOLS have reduced genetic variation in this breed, which may have allowed detecting significant differences according to the diet. These hypotheses or a combination of these may partly explain the absence of significant modification of miRNA gene expression. Thus, the following results and discussion are specific to Holstein breed.

**Table 3:** Differentially expressed miRNAs in the mammary gland of midlactation Holstein cows during restriction (REST) compared to the control (CONT) period.

Name	Mean read		Expression ratio	FDR	Expression change
	CONT	REST			
***bta-miR-143***	**2,292,707.1**	**3,119,812.5**	**1.37**	**1.4E-02**	**Up**
***bta-miR-181a***	**197,294.7**	**270,887.9**	**1.37**	**2.9E-02**	**Up**
***bta-miR-26b***	**97,181.9**	**138,114.2**	**1.43**	**1.4E-02**	**Up**
***bta-miR-200c***	**65,416.1**	**48,881.0**	**0.75**	**3.6E-02**	**Down**
***bta-miR-25***	**19,640.0**	**24,430.3**	**1.25**	**1.7E-02**	**Up**
***bta-miR-200b***	**17,718.1**	**12,055.1**	**0.68**	**1.2E-02**	**Down**
***bta-miR-181b***	**8,203.8**	**11,208.3**	**1.37**	**2.6E-02**	**Up**
***bta-miR-155***	**1,178.0**	**1,724.1**	**1.47**	**3.9E-02**	**Up**
*bta-miR-193a-3p*	889.5	597.8	0.67	2.2E-06	Down
*bta-miR-669*	695.1	537.3	0.78	2.6E-02	Down
*bta-miR-374b*	694.3	533.5	0.77	9.3E-03	Down
*bta-miR-379*	377.7	526.7	1.39	5.3E-02	Up
*bta-miR-222*	266.7	370.5	1.39	2.2E-03	Up
*bta-let-7a-3p*	348.6	207.4	0.60	3.3E-03	Down
*bta-miR-326*	198.5	138.9	0.70	5.8E-03	Down
*bta-miR-2898*	199.2	117.8	0.59	5.8E-03	Down
*bta-miR-500*	174.1	104.1	0.60	9.3E-03	Down
*bta-miR-29d-5p*	98.8	67.1	0.68	3.5E-02	Down
*bta-miR-495*	70.5	26.2	0.37	1.3E-08	Down
*bta-miR-299*	74.9	19.4	0.26	1.3E-08	Down
*bta-miR-299-2*	71.9	17.7	0.25	1.3E-08	Down
*bta-miR-671*	50.8	28.4	0.56	5.8E-03	Down
chr7_8584_mature	55.5	17.5	0.32	6.9E-04	Down
*bta-miR-296-5p*	47.8	25.0	0.52	9.5E-03	Down
chr10_11366_mature	31.3	12.6	0.40	3.5E-03	Down
*bta-miR-18b*	29.3	12.5	0.44	1.4E-02	Down
*bta-miR-2483-5p*	17.2	6.6	0.40	2.4E-02	Down

Means correspond to the mean of normalized read counts. The bold and italic line corresponds to all miRNAs with more than 1,000 reads and known miRNAs, respectively. FDR: False discovery rate.

Among the 27 DEMs affected by the restriction in HOLS, 25 are known and 2 predicted with 19 down- and 8 upregulated by feed restriction. Eight DEMs were highly expressed with more than 1,000 reads (Table 3). Thirty DEMs were modulated by 48 h of feed deprivation in the MG of lactating goats [10]. The number of DEMs in the current study is similar to those reported in the study of Mobuchon et al. [10] using similar analyses expected that the cut-off was 0.1 in the study performed by Mobuchon et al. and 0.05 in the present study. Two miRNAs are common to our study: *miR-222*, which was upregulated with a similar fold change (**FC**) between studies (FC: 1.47 vs 1.39 in caprine and bovine, respectively), and *miR-671*, which was downregulated (FC: 0.73 vs 0.56 in caprine and bovine, respectively). However, whereas *miR-671* presented a similar level of expression in bovine and caprine MG, *miR-222* showed more reads in caprine (812 during control and 1,408 in feed deprived goats) than in bovine (267 in CONT and 371 in REST), suggesting a higher level of *miR-222* expression in caprine MG compared to bovine.

Early lactation is known to be a natural period of NEB for dairy ruminants. A comparison of DEMs obtained during an induced NEB in cows (present study) and during early lactation in goats [30] highlighted similarities between these two species. For instance, Ji et al. [30] observed 378 DEMs in goat MG between early and late lactation periods using all mammalian miRNA sequences for mapping. Among them, 4 DEMs were common to the present study: *miR-181a* and *-299*, *-2483-5P* (mapped on ruminant genome) and *miR*-200c (mapped on Tasmanian devil [30]). Except for *miR-200c*, all other miRNAs presented a similar manner of regulation as our study. Among the common miRNAs, *miR-181a* and miR*-200c* are highly expressed miRNAs in our study. Wang et al. [7] showed that the expression of 13 miRNAs in bovine MG was greater during NEB (i.e., early lactation) than during the positive energy balance period (i.e., dry period). Despite the dissimilarity of physiological states between Wang et al. [7] (early vs non-lactation period) and our study, two differentially expressed miRNAs (*miR-155* and *-181a*) are common and have a similar sense of regulation by energy balance. These miRNAs belong to the highly expressed DEMs in our study.

#### Differentially expressed miRNAs located in milk QTL

In cattle, at least 50,178 quantitative trait loci (QTL) out of a total of 130,471 have been associated with milk production traits (https://www.animalgenome.org). Analyses of genome positions of the 25 known DEMs in the current study revealed that 8 DEMs are associated with a milk QTL (Table 4). One (*miR*-155) was within a QTL associated with milk α-lactalbumin content, and 7 miRNAs were associated with milk fat composition (Table 4). Among these 7 miRNAs, 5 (*miR-200c*, *-2898*, *-181a*, *-181b*, and *-296-5p*) were located in QTL associated with saturated fatty acid content, and 4 (*miR-26b*, *-200c*, *-296-5p*, and *-669*) were located in QTL linked to monounsaturated fatty acid content. The difference in the number of miRNAs observed in QTL of saturated vs. unsaturated fatty acids may be due to the difference in the abundance of QTL associated with two classes of fatty acids [31]. In bovines, the close genome positions of *miR-181a* and *miR-181b* (< 10 kb; referred to miRBase criteria) on chromosome 11 suggest that they are in a cluster. This finding is in line with the results in mice reporting that they belong to the same family and are in a cluster [32]. Their cluster is located within the same QTL associated with the regulation of butyric and caproic acid content in milk (Table 4). Moreover, the *miR-181a* and *miR-181b* upregulation and their high level of expression during the REST period may be related, at least partly, to the decrease in short chain fatty acid content. Indeed, in our study, ∑C4 to C9 was 5.41 vs 3.43 g/100 g total fatty acids during CONT and REST (S1 Table), respectively (P < 0.001).

**Table 4:** Location of nutriregulated miRNAs in QTL associated with milk production and composition.

miRNA name	Chromosome	Start	End	Character
*bta-miR-155*	1	10227315	10227337	Milk alpha-lactalbumin percentage
*bta-miR-26b*	2	107133408	107133429	Milk oleic acid content
*bta-miR-200c*	5	103859438	103859460	Milk myristic acid content; Milk palmitoleic acid content
*bta-miR-2898*	8	74354027	74354044	Milk capric acid content; Milk myristic acid content
*bta-miR-181a*	11	95709449	95709472	Milk butyric acid content; Milk caproic acid content
*bta-miR-181b*	11	95710641	95710664	Milk butyric acid content; Milk caproic acid content
*bta-miR-296-5p*	13	58091948	58091966	Milk capric acid content; Milk caproic acid content; Milk caprylic acid content; Milk myristoleic acid content; Milk palmitoleic acid content
*bta-miR-669*	16	63239604	63239625	Milk palmitoleic acid content

Characters are from https://www.animalgenome.org.

#### Analyses of the 8 highly expressed and differential miRNAs

We focused on highly expressed miRNAs to decipher the mechanisms underlying the response of MG to restriction. Among the 25 known DEMs in Holstein cows, 8 (*miR-143*, *miR-181a*, *miR-26b*, *miR-200c*, *miR-25*, *miR-200b*, *miR-181b*, and *miR-155*) presented more than 1,000 reads, representing 99% of the total reads of the DEMs (Table 3). *MiR-143* was the most abundant miRNA in this study, possessing more than 2 million reads, which is in agreement with previous studies in bovine MG [33] and bovine liver [34, 35]. *MiR-143*, known to regulate lipid metabolism, was downregulated in bovine liver during severe NEB [34]. In the present study, *miR-143* was upregulated in MG during the NEB period. This difference in regulation may be due to the studied tissue (liver vs MG). Indeed, the NEB period is characterized by strong adipose tissue mobilization, increased NEFA esterification, triacylglycerol accumulation, and ketone synthesis in the liver, as well as decreased lipid metabolism in the MG [36].

*MiR-26b* and *-181a* are, respectively, the second and third most expressed miRNAs in our study, possessing more than 100,000 reads. *MiR-26b* was identified as playing a role in inflammation [37]. The upregulation of *miR-26b* in our study might be linked to inflammation due to restriction. Indeed, lipid mobilization during NEB may lead to inflammation [38]. *MiR-181a* was reported to influence lipid and glucose metabolism in humans, mice and bovine liver [39]. *MiR-181a* targets *ACSL1* mRNA, which is involved in lipid synthesis in bovine mammary epithelial cells [13]. The upregulation of *miR-181a* in our study may be related to the decrease in milk fat yield observed during the NEB period (Table 1) and the diminution of short- (data not shown) and medium-chain fatty acids [18]. Ma et al. [40] showed that *miR-25* was involved in triacylglycerol synthesis and lipid accumulation in goat mammary epithelial cells. This miRNA directly targets *PPARG* and regulates the expression of *SREBP1*, *FASN*, and *GPAM* involved in lipid synthesis. The observed upregulation of *miR-181a* and *miR-25* may be related to the decrease in fat yield during the feed restriction period. Among the highly expressed DEMs, *miR-200b* and *miR-200c* belong to the same family, have the same seed sequence, and regulate epithelial-to-mesenchymal transition in breast cancer [41, 42]. The downregulation of *miR-200b* and *miR-200c* in our study may suggest a link with MG structural changes due to feed restriction [43].

*MiR-181b* and *miR-155* were the least expressed of the 8 miRNAs and were upregulated by feed restriction. The modulation of their expression may be linked to the stress induced by feed restriction. Indeed, *miR-181b* was upregulated in the serum of heat-stressed Frieswal bulls compared with summer and winter periods [44] and after chirurgical stress in Angus cattle muscle [45]. In addition, *miR-181b* and *miR-155* were also reported to play a role in the immune response [46, 47]. LPS stimulation of mouse macrophages *in vitro* resulted in the upregulation of *miR-155*, and similar changes also occurred after intraperitoneal LPS injection in C57BL/6 mice [47]. In addition, *miR-155* promoted the production of the proinflammatory cytokine TNF-α in human B cells [47]. Thus, the upregulation of *miR-181b* and *miR-155* observed in the present study may be explained by inflammation that can be affected by NEB, as demonstrated in other studies [38].

### Differentially expressed mRNAs in Holstein cows

To elucidate the mechanisms of nutritional regulation of MG function, mRNA analysis was performed. Considering the results obtained by miRNome studies, mRNA analysis was achieved only in HOLS cows. Microarray analyses identified 374 differentially expressed genes (**DEGs**, P_adj_ ≤0.1) between the CONT and REST periods with 120 upregulated and 254 downregulated genes during the REST period. Ten DEGs presented an FC ≥ 3. In caprine MG, Ollier et al. [2] identified 161 DEGs whose expression was altered by 48 h of feed deprivation using the same bovine microarray. The lower number of DEGs reported by Ollier et al. [2] might be due to the heterogeneous system used (caprine mRNA with a bovine array). Bioinformatic analysis of the 374 DEGs showed 256 altered biological processes (**BPs**; P ≤ 0.05). The analyses of the first 25 BPs revealed that 12 BPs were linked to lipid metabolism and involved 28 DEGs (23 downregulated and 5 upregulated), and 9 BPs were related to respiration metabolism and involved 33 DEGs (30 downregulated and 3 upregulated; Table 5). In addition, metabolic process (S2 Table) and SCAP/SREBP, cholesterol biosynthesis and regulation of lipid metabolism (S3 Table) were also highlighted using Metacore^TM^ analysis. Despite the fact that the results were corroborated between different bioinformatic analyses, it should be noted that a limitation of these algorithms is that they did not consider the background corresponding to the exact gene representation of the whole genome microarray used.

**Table 5:** Top 25 biological processes potentially regulated by the 374 DEGs in Holstein cows.

		Term	Overlap	P-value	FDR
%	1	mitochondrial ATP synthesis coupled electron transport	27/86	4.7E-26	8.9E-23
%	2	respiratory electron transport chain	27/95	9.6E-25	9.1E-22
%	3	mitochondrial electron transport, NADH to ubiquinone	21/47	2.2E-24	1.4E-21
%	4	mitochondrial respiratory chain complex I biogenesis	20/65	2.1E-19	6.5E-17
%	5	NADH dehydrogenase complex assembly	20/65	2.1E-19	6.5E-17
%	6	mitochondrial respiratory chain complex I assembly	20/65	2.1E-19	6.5E-17
%	7	mitochondrial respiratory chain complex assembly	22/98	5.9E-18	1.6E-15
*	8	cholesterol biosynthetic process	11/36	3.2E-11	7.7E-09
*	9	sterol biosynthetic process	11/41	1.6E-10	3.3E-08
	10	secondary alcohol biosynthetic process	10/37	1.0E-09	1.9E-07
*	11	regulation of cholesterol biosynthetic process	9/41	5.1E-08	8.8E-06
*	12	regulation of cholesterol metabolic process	9/42	6.4E-08	1.0E-05
	13	regulation of alcohol biosynthetic process	8/35	2.1E-07	3.0E-05
*	14	long-chain fatty acid transport	7/26	3.6E-07	4.9E-05
*	15	cholesterol metabolic process	10/69	5.8E-07	7.3E-05
	16	regulation of steroid biosynthetic process	8/45	1.6E-06	1.9E-04
*	17	sterol metabolic process	8/60	1.5E-05	1.7E-03
%	18	mitochondrial transmembrane transport	6/33	2.9E-05	3.1E-03
*	19	fatty acid metabolic process	10/107	3.2E-05	3.2E-03
	20	carnitine shuttle	4/12	5.2E-05	4.7E-03
*	21	fatty acid transmembrane transport	4/12	5.2E-05	4.7E-03
*	22	lipid biosynthetic process	8/73	6.3E-05	5.5E-03
%	23	cellular respiration	7/58	9.8E-05	8.1E-03
*	24	intracellular lipid transport	4/16	1.8E-04	1.4E-02
*	25	fatty-acyl-CoA biosynthetic process	5/31	2.5E-04	1.9E-02

Process network analysis was performed using Enrichr software.

% indicates processes associated with respiration, and * indicates lipid metabolism processes. The overlap corresponds to the ratio of the DEG number on the number of genes known in the BP. FDR: false discovery rate.

**Fig 1:**
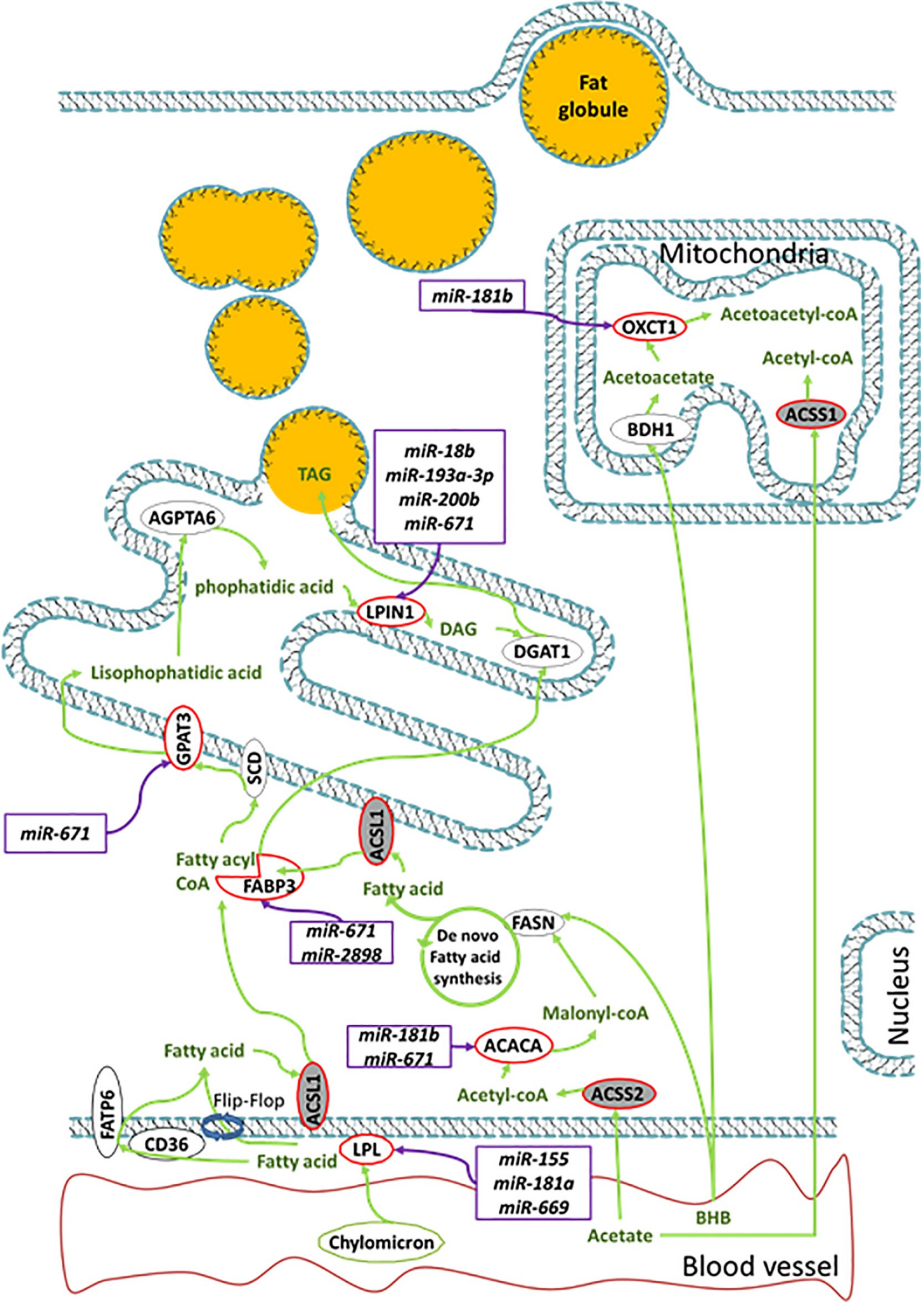
MiRNAs and coding genes involved in the milk fat synthesis pathway. Genes in the red circle correspond to DEGs identified in our study, and miRNAs in the purple box correspond to DEMs targeting the DEGs (adapted from Bionaz and Loor [52]).

The effects of feed restriction on genes involved in lipid metabolism in the MG of lactating Holstein cows (Fig 1) were in agreement with previous studies. The downregulation of *ACACA*, *FABP3* and *LPL* in our study was in accordance with the reduction of their expression after 4 days of restriction (cows fed at 60% of their *ad libitum* DMI; [48]). The downregulation of *ACSL1*, *FABP3*, and *LPIN1* in mammary tissue of dairy cows is in line with the decrease in their expression during the natural NEB period of early lactation (day 15 of lactation) compared to midlactation (day 60 of lactation), which is associated with a lower fat yield [49]. Taken together, these results indicate that the downregulation of the DEGs (*ACACA*, *FABP3*, *LPL*, *LPIN1* and *ACSL1*) involved in lipid metabolism observed in our study (Fig 1) could be related to the decrease in milk fat yield observed during REST.

In the present study, feed restriction downregulated 21 DEGs (e.g., *NDUFA3*, *NDUFB1*, and *NDUFS2*) which are related to the NADH pathway and respiration (Table 5, S2 Table). A downregulation of several NADH dehydrogenase mRNAs was previously observed in bovine liver during heat stress [50], suggesting that feed restriction may affect energy production through mitochondrial respiration, which was already identified among the most inhibited functions in bovine liver [51].

### Analysis of mRNAs common between the DEG list and mRNAs targeted by the 8 major DEMs

**Fig 2:**
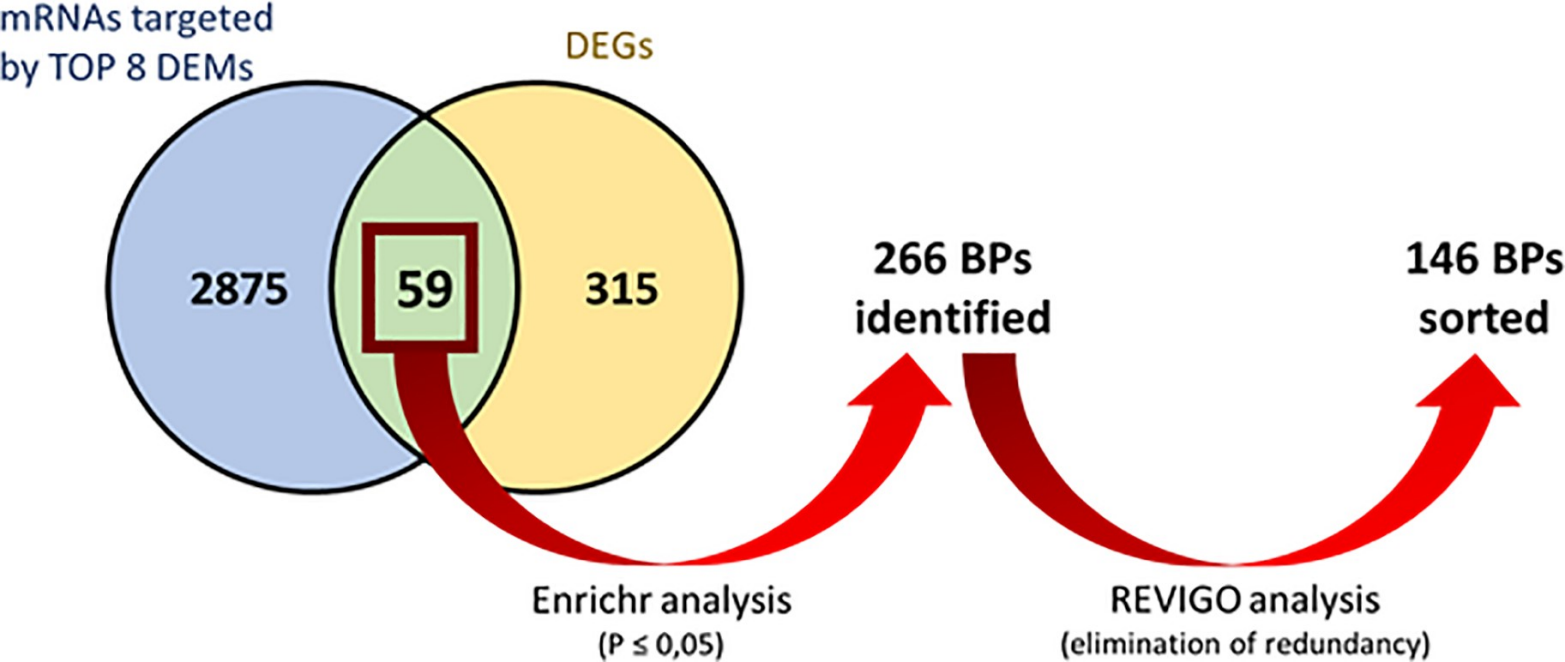
Comparison between mRNA targeted by the 8 most expressed DEMs and DEGs in Holstein cows. Venn diagram between the potential targets of DEMs (blue circle) and the 374 DEGs (yellow circle). Red arrows indicate the workflow used to analyze biological processes (BPs) common mRNA with the Enrichr and REVIGO platforms.

**Fig 3:**
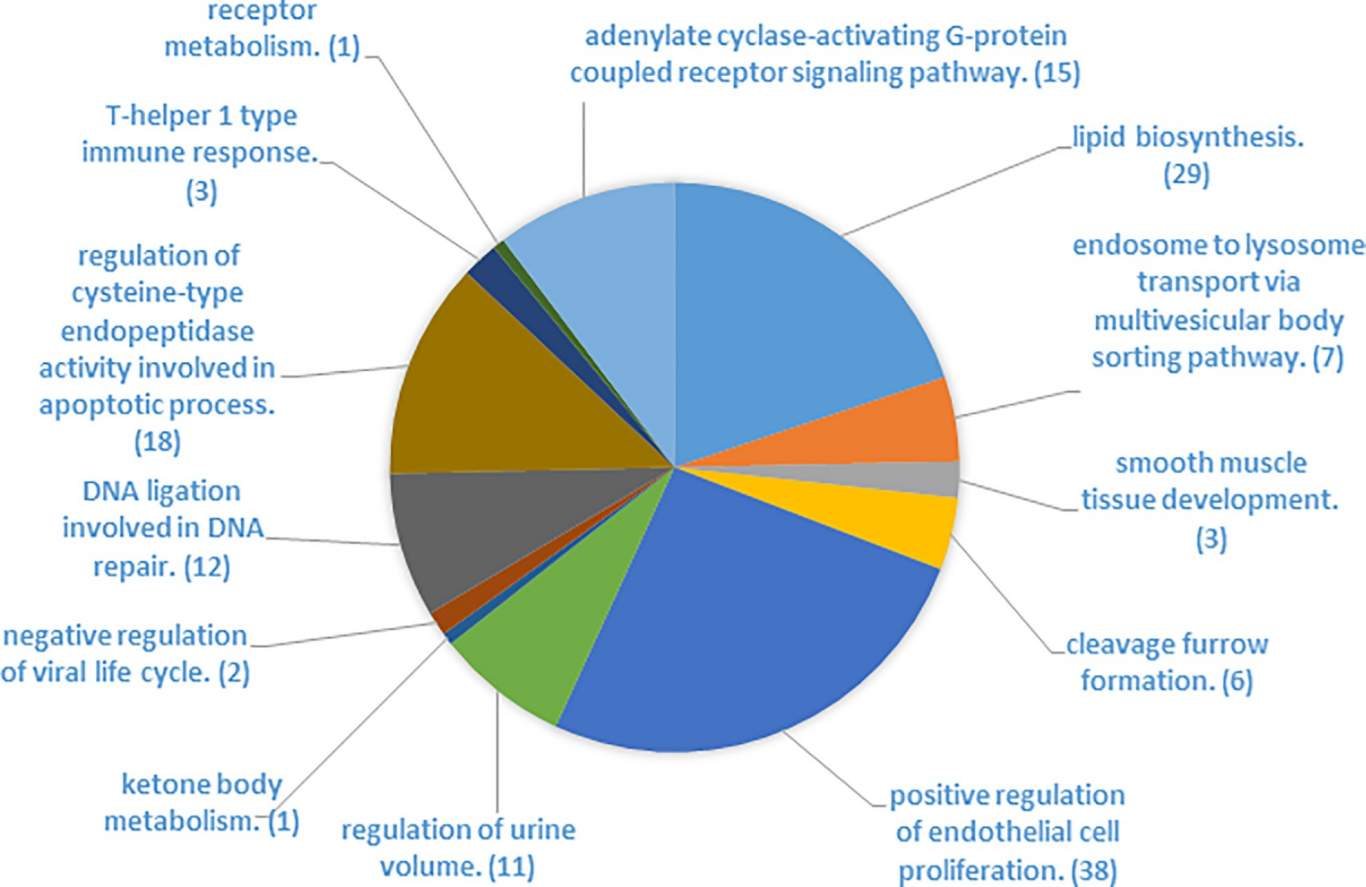
REVIGO categories obtained from the Enrichr biological process list using the 59 common mRNAs (P ≤ 0.05). In brackets: Numbers of BPs in each category.

Using miRWalk analyses, we identified 2934 mRNAs potentially targeted by the 8 most expressed DEMs in Holstein cows. A comparison between those potential targets and the 374 DEGs identified by microarray analysis showed 59 common mRNAs (Fig 2). Among these mRNAs, 37 were downregulated, and 22 were upregulated. Two DEGs (*LPIN1* and *LPL*) had an absolute FC ≥ 3, and both were downregulated. *LPIN1* and *LPL* are involved in lipid metabolism [48, 49]. Analysis of the 59 common mRNAs by the Enrichr platform identified 266 BPs (P ≤ 0.05). The elimination of redundancy using the REVIGO platform highlighted 146 BPs, which were regrouped into 13 categories denoting a semantic similarity between BPs (Figs 2 and 3), and 6 of these categories represented 89% of BPs. Two categories (positive regulation of endothelial cell proliferation and lipid biosynthesis) contained more than 20% BPs each. Positive regulation of endothelial cell proliferation was the first category, with 38 of the 146 BPs being regulated. Among those 38 BP, 19 are predicted to be regulated by PROX1 (Prospero Homeobox 1; S4 Table), which is a downregulated DEG that is targeted *by miR-181a*, a highly expressed miRNA that was determined to be upregulated by REST in HOLS cows. PROX1 is a transcriptional factor influencing cell fate, gene transcriptional regulation and progenitor cell regulation, playing a role in lymphatic system development. Wilge and Oliver [53] showed that knockouts in *PROX1* mouse embryos lacked lymphatic system development. Elsewhere, a decrease in lymphatic vasculature (identified using *PROX1* and *CD31*) in MG was observed across the lactation of mice [54]. In our study, the upregulation of *miR-181a* and the downregulation of *PROX1* by feed restriction suggest a modification of lymphatic vasculature. In addition, *PROX1* was hosted in a QTL associated with milk palmitoleic acid content and cheese-making properties [55]. The modulation of *miR-181a* and *PROX1* gene expression is consistent with the milk fatty acid changes observed during feed restriction [18].

Lipid biosynthesis had 29 of the 146 BPs and was the second category identified by REVIGO analysis. Furthermore, three BPs (lipid biosynthetic process, triglyceride biosynthetic process and fatty acid metabolic process) were also identified among the top 15 BPs identified by Enrichr analyses. In these three BPs, four DEGs (*ACACA*, *CYP51A1*, *LPL*, and *LPIN1*) were downregulated by restriction. A downregulation of *CYP51A1*, a gene involved in cholesterol synthesis, was already reported during feed restriction in chicken liver [56] and in adipose tissue of beef cows [57]. *ACACA*, *CYP51A1*, *LPL*, and *LPIN1* are predicted to be targeted by 4 DEMs (*miR-155*, *-181a*, *-181b*, and *-200b*). *MiR-181a* and *miR-155* target *LPL*, *miR-181b* target *ACACA*, and *miR-200b* target *LPIN1* and *CYP51A1*. The upregulation of *miR-181a*, *-181b*, and *-155* was in line with the downregulation of their targeted genes. A study demonstrated that the absence of *miR-155* led to an increase in *LPL* expression in the liver of *miR-155*^-/-^ mice [58].

## Conclusions

Feed restriction modified MG miRNome and transcriptome in Holstein but not in Montbéliarde cows. The differentially expressed miRNAs highlight a potential role of miRNAs in MG remodeling and lipid synthesis. Moreover, these roles were supported by the analyses of common differential mRNAs and predicted targets of miRNAs sharing the same function. In addition, the location of differentially expressed miRNAs in QTL associated with milk fatty acid composition was consistent with the observed reduction in short- and medium-chain milk fatty acid content in milk. The decrease in *de novo* milk fatty acid synthesis observed during restriction mediated in part by the regulation of the miRNAs affecting mRNAs involved in lipid metabolism and suggest a role of DEMs in this biological process.

## Supporting information

S1 Fig Homemade bioinformatics script in Python language for screening QTL. (TIF)

S1 Table Effects of feed restriction on milk fatty acid concentrations (g/100 g of FA) in mid-lactation cows. (DOCX)

S2 Table TOP 20 of the biological processes affected by restriction in mammary gland of mid-lactation cows. Enrichment by GO Processes using Metacoret^TM^ software using the 374 differentially expressed genes after restriction. FDR: false Discovery Rate.
(DOCX)

S3 Table TOP 20 of the pathways affected by restriction in mammary gland of mid-lactation cows. Enrichment by Pathway maps using Metacoret^TM^ software using the 374 differentially expressed genes after restriction. FDR: false Discovery Rate.
(DOCX)

S4 Table Biological processes predicted to be regulated by Prox1 identified by bioinformatics analyses of the 59 genes differentially expressed and predicted to be targeted by the 8 major differentially expressed miRNAs highlighted the regulation of endothelial cell proliferation containing 38 biological processes among which 19 were predicted to be regulated by Prox1. (DOCX)
