# Supplementary figures and images for: Nutrigenomic analyses reveal miRNAs and mRNAs affected by feed restriction in the mammary gland of midlactation dairy cows
Source: PLoS One. 2021 Apr 15;16(4):e0248680. doi: 10.1371/journal.pone.0248680 (PMC8049318; doi:10.1371/journal.pone.0248680)

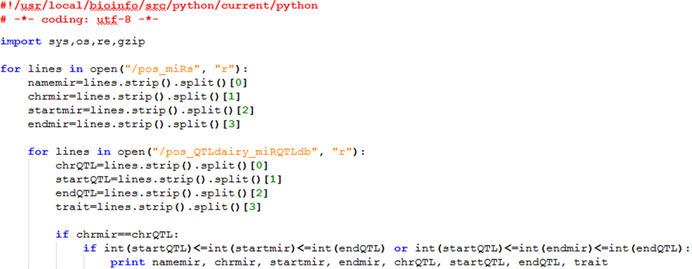

Supplement: S1 Fig — (TIF) [file pone.0248680.s001.tif]
