# Supplementary material for: Nutrigenomic analyses reveal miRNAs and mRNAs affected by feed restriction in the mammary gland of midlactation dairy cows
Source: PLoS One. 2021 Apr 15;16(4):e0248680. doi: 10.1371/journal.pone.0248680 (PMC8049318; doi:10.1371/journal.pone.0248680)
Supplement: S1 Table — (DOCX) [file pone.0248680.s002.docx]

**S1 Table**: **Effects of feed restriction on milk fatty acid concentrations (g/100 g of FA) in mid-lactation cows.**

|  | CONT | REST | SEM | p value |
| --- | --- | --- | --- | --- |
| C4 | 1,45 | 1,25 | 0,07 | 0,065 |
| C5 | 0,02 | 0,01 | 0,00 | 0,004 |
| C6 | 2,44 | 1,47 | 0,10 | 0,000 |
| C7 | 0,04 | 0,01 | 0,00 | 0,002 |
| C8 | 1,42 | 0,68 | 0,05 | <.0001 |
| C9 | 0,05 | 0,01 | 0,01 | 0,004 |
| C10 | 3,26 | 1,22 | 0,10 | 0,0001 |
| C10:1 cis9 | 0,37 | 0,14 | 0,01 | 0,0001 |
| C12 | 4,04 | 1,38 | 0,15 | <.0001 |
| C14 | 12,82 | 6,39 | 0,27 | <.0001 |
| C14:1 cis9 | 1,48 | 0,57 | 0,05 | <.0001 |
| C15 | 1,32 | 0,73 | 0,09 | 0,0022 |
| C16 | 36,95 | 23,92 | 0,78 | 0,0001 |
| C18 | 6,41 | 11,47 | 0,42 | 0,0004 |
| C181 cis9 | 15,90 | 34,71 | 0,75 | <.0001 |
|  |  |  |  |  |
| Σ C4 to C9 | 5,41 | 3,43 | 0,20 | 0,0001 |
| Σ C4 to C15 | 29,88 | 14,69 | 0,66 | <.0001 |
| Σ C10 to C15 | 24,50 | 11,27 | 0,54 | <.0001 |
| Σ >C16 | 30,46 | 58,36 | 0,94 | <.0001 |
|  |  |  |  |  |
