# Supplementary material for: Nutrigenomic analyses reveal miRNAs and mRNAs affected by feed restriction in the mammary gland of midlactation dairy cows
Source: PLoS One. 2021 Apr 15;16(4):e0248680. doi: 10.1371/journal.pone.0248680 (PMC8049318; doi:10.1371/journal.pone.0248680)
Supplement: S2 Table — Enrichment by GO Processes using MetacoretTM software using the 374 differentially expressed genes after restriction. FDR: false Discovery Rate. (DOCX) [file pone.0248680.s003.docx]

**S2 Table: TOP 20 of the biological processes affected by restriction in mammary gland of mid-lactation cows.** Enrichment by GO Processes using Metacoret^TM^ software using the 374 differentially expressed genes after restriction. FDR: false Discovery Rate

| **Processes** | **Total** | **pValue** | **FDR** | **In data** |
| --- | --- | --- | --- | --- |
| [mitochondrial ATP synthesis coupled electron transport](http://portal.genego.com/cgi/process.cgi?id=-1614472597) | 111 | 2,229E-30 | 6,352E-27 | 32 |
| [oxidative phosphorylation](http://portal.genego.com/cgi/process.cgi?id=-1038170157) | 144 | 2,787E-30 | 6,352E-27 | 35 |
| [ATP synthesis coupled electron transport](http://portal.genego.com/cgi/process.cgi?id=-1519345543) | 112 | 3,073E-30 | 6,352E-27 | 32 |
| [respiratory electron transport chain](http://portal.genego.com/cgi/process.cgi?id=-1588345901) | 137 | 1,801E-28 | 2,793E-25 | 33 |
| [mitochondrial electron transport, NADH to ubiquinone](http://portal.genego.com/cgi/process.cgi?id=-598716027) | 54 | 9,369E-27 | 1,162E-23 | 23 |
| [electron transport chain](http://portal.genego.com/cgi/process.cgi?id=-673513725) | 232 | 4,739E-26 | 4,898E-23 | 38 |
| [cellular respiration](http://portal.genego.com/cgi/process.cgi?id=-208392230) | 223 | 1,346E-25 | 1,193E-22 | 37 |
| [metabolic process](http://portal.genego.com/cgi/process.cgi?id=-412825077) | 10691 | 2,182E-25 | 1,691E-22 | 288 |
| [cellular metabolic process](http://portal.genego.com/cgi/process.cgi?id=-203083230) | 9623 | 4,405E-25 | 3,036E-22 | 269 |
| [generation of precursor metabolites and energy](http://portal.genego.com/cgi/process.cgi?id=-1029783272) | 557 | 1,399E-22 | 8,675E-20 | 51 |
| [mitochondrial respiratory chain complex I assembly](http://portal.genego.com/cgi/process.cgi?id=-1632798087) | 72 | 1,828E-20 | 9,446E-18 | 21 |
| [NADH dehydrogenase complex assembly](http://portal.genego.com/cgi/process.cgi?id=-670801768) | 72 | 1,828E-20 | 9,446E-18 | 21 |
| [cofactor metabolic process](http://portal.genego.com/cgi/process.cgi?id=-21819000) | 603 | 2,815E-20 | 1,343E-17 | 50 |
| [energy derivation by oxidation of organic compounds](http://portal.genego.com/cgi/process.cgi?id=-289866600) | 341 | 5,787E-20 | 2,564E-17 | 38 |
| [oxidation-reduction process](http://portal.genego.com/cgi/process.cgi?id=-400807333) | 1245 | 8,464E-20 | 3,500E-17 | 72 |
| [cofactor biosynthetic process](http://portal.genego.com/cgi/process.cgi?id=-1640856334) | 245 | 4,790E-19 | 1,857E-16 | 32 |
| [mitochondrial respiratory chain complex assembly](http://portal.genego.com/cgi/process.cgi?id=-1202882968) | 112 | 1,605E-18 | 5,855E-16 | 23 |
| [small molecule metabolic process](http://portal.genego.com/cgi/process.cgi?id=-1070727628) | 2020 | 8,515E-18 | 2,934E-15 | 91 |
| [response to drug](http://portal.genego.com/cgi/process.cgi?id=-516598693) | 1915 | 3,222E-16 | 1,052E-13 | 85 |
| [phosphate-containing compound metabolic process](http://portal.genego.com/cgi/process.cgi?id=-968658357) | 2455 | 8,080E-15 | 2,448E-12 | 96 |
