# Supplementary material for: Nutrigenomic analyses reveal miRNAs and mRNAs affected by feed restriction in the mammary gland of midlactation dairy cows
Source: PLoS One. 2021 Apr 15;16(4):e0248680. doi: 10.1371/journal.pone.0248680 (PMC8049318; doi:10.1371/journal.pone.0248680)
Supplement: S3 Table — Enrichment by Pathway maps using MetacoretTM software using the 374 differentially expressed genes after restriction. FDR: false Discovery Rate. (DOCX) [file pone.0248680.s004.docx]

**S3 Table: TOP 20 of the pathways affected by restriction in mammary gland of mid-lactation cows.** Enrichment by Pathway maps using Metacoret^TM^ software using the 374 differentially expressed genes after restriction. FDR: false Discovery Rate

| **Pathway maps** | **Total** | **pValue** | **FDR** | **In data** |
| --- | --- | --- | --- | --- |
| [Ubiquinone metabolism](http://portal.genego.com/cgi/imagemap.cgi?id=884) | 74 | 3,830E-21 | 3,792E-18 | 22 |
| [Immune response_Lectin induced complement pathway](http://portal.genego.com/cgi/imagemap.cgi?id=665) | 50 | 3,257E-06 | 1,269E-03 | 8 |
| [Immune response_Classical complement pathway](http://portal.genego.com/cgi/imagemap.cgi?id=477) | 53 | 5,127E-06 | 1,269E-03 | 8 |
| [Immune response_Alternative complement pathway](http://portal.genego.com/cgi/imagemap.cgi?id=476) | 53 | 5,127E-06 | 1,269E-03 | 8 |
| [SCAP/SREBP Transcriptional Control of Cholesterol and FA Biosynthesis](http://portal.genego.com/cgi/imagemap.cgi?id=2782) | 45 | 1,653E-05 | 3,244E-03 | 7 |
| [Cholesterol Biosynthesis](http://portal.genego.com/cgi/imagemap.cgi?id=835) | 103 | 1,966E-05 | 3,244E-03 | 10 |
| [Alternative complement cascade disruption in age-related macular degeneration](http://portal.genego.com/cgi/imagemap.cgi?id=7234) | 31 | 2,347E-04 | 3,319E-02 | 5 |
| [G protein-coupled receptors signaling in lung cancer](http://portal.genego.com/cgi/imagemap.cgi?id=6201) | 76 | 4,913E-04 | 6,080E-02 | 7 |
| [MAPK-mediated proliferation of normal and asthmatic smooth muscle cells](http://portal.genego.com/cgi/imagemap.cgi?id=5301) | 56 | 5,541E-04 | 6,095E-02 | 6 |
| [Complement pathway disruption in thrombotic microangiopathy](http://portal.genego.com/cgi/imagemap.cgi?id=6612) | 39 | 7,055E-04 | 6,985E-02 | 5 |
| [Retinal ganglion cell damage in glaucoma](http://portal.genego.com/cgi/imagemap.cgi?id=7233) | 45 | 1,369E-03 | 1,129E-01 | 5 |
| [Signal transduction_Angiotensin II/ AGTR1 signaling via JAK/STAT](http://portal.genego.com/cgi/imagemap.cgi?id=439) | 45 | 1,369E-03 | 1,129E-01 | 5 |
| [Regulation of lipid metabolism_PPAR regulation of lipid metabolism](http://portal.genego.com/cgi/imagemap.cgi?id=632) | 47 | 1,668E-03 | 1,270E-01 | 5 |
| [Development_EDNRB signaling](http://portal.genego.com/cgi/imagemap.cgi?id=2273) | 50 | 2,204E-03 | 1,558E-01 | 5 |
| [Stem cells_Role of Ceramide 1-phosphate, Sphingosine 1-phosphate and Complement cascade in hematopoietic stem cell homing](http://portal.genego.com/cgi/imagemap.cgi?id=6423) | 32 | 2,704E-03 | 1,785E-01 | 4 |
| [Chemotaxis_CCL19- and CCL21-mediated chemotaxis](http://portal.genego.com/cgi/imagemap.cgi?id=7043) | 33 | 3,033E-03 | 1,877E-01 | 4 |
| [Regulation of Beta-catenin activity in colorectal cancer](http://portal.genego.com/cgi/imagemap.cgi?id=6458) | 56 | 3,634E-03 | 2,116E-01 | 5 |
| [Propionate metabolism p.1](http://portal.genego.com/cgi/imagemap.cgi?id=823) | 36 | 4,182E-03 | 2,300E-01 | 4 |
| [A shift from oxidative to glycolytic muscle fiber phenotype in quadriceps muscles in COPD](http://portal.genego.com/cgi/imagemap.cgi?id=6673) | 60 | 4,897E-03 | 2,309E-01 | 5 |
| [Neutrophil resistance to apoptosis in COPD and proresolving impact of lipid mediators](http://portal.genego.com/cgi/imagemap.cgi?id=6494) | 60 | 4,897E-03 | 2,309E-01 | 5 |
