# Supplementary material for: Nutrigenomic analyses reveal miRNAs and mRNAs affected by feed restriction in the mammary gland of midlactation dairy cows
Source: PLoS One. 2021 Apr 15;16(4):e0248680. doi: 10.1371/journal.pone.0248680 (PMC8049318; doi:10.1371/journal.pone.0248680)
Supplement: S4 Table — (DOCX) [file pone.0248680.s005.docx]

**S4 Table**: **Biological processes predicted to be regulated by Prox1** identified by bioinformatics analyses of the 59 genes differentially expressed and predicted to be targeted by the 8 major differentially expressed miRNAs highlighted the regulation of endothelial cell proliferation containing 38 biological processes among which 19 were predicted to be regulated by Prox1.

| ***Description*** | ***log10pvalue*** | ***Gene symbol*** | ***with Prox1*** |
| --- | --- | --- | --- |
| Positive regulation of endothelial cell proliferation | -300 | **APLNR; PROX1; HMGB1** | X |
| Positive regulation of vascular endothelial cell proliferation | -1,5229 | HMGB1 |  |
| Positive regulation of cell development | -300 | MYOD1; **PROX1** | X |
| Coronary vasculature development | -1,699 | APLNR |  |
| Apoptotic DNA fragmentation | -1,301 | HMGB1 |  |
| T-helper cell differentiation | -1,5229 | HMGB1 |  |
| Cardiocyte differentiation | -1,3979 | **PROX1** | X |
| Regulation of RNA polymerase II transcriptional preinitiation complex assembly | -1,5229 | HMGB1 |  |
| Regulation of dendritic cell differentiation | -1,699 | HMGB1 |  |
| Negative regulation of dendrite morphogenesis | -1,699 | EFNA1 |  |
| Aorta morphogenesis | -1,301 | **PROX1** | X |
| Lens fiber cell development | -1,699 | **PROX1** | X |
| Nose development | -1,5229 | **PROX1** | X |
| Regulation of neural precursor cell proliferation | -1,3979 | **PROX1** | X |
| Cell morphogenesis involved in differentiation | -2 | EFNA1; **PROX1** | X |
| Cell differentiation in hindbrain | -1,3979 | **PROX1** | X |
| Muscle cell fate commitment | -1,5229 | MYOD1 |  |
| Central nervous system projection neuron axonogenesis | -1,5229 | PAFAH1B1 |  |
| Positive regulation of supramolecular fiber organization | -1,5229 | KATNB1; **PROX1** | X |
| Vasculogenesis | -300 | APLNR; ADM |  |
| Venous blood vessel morphogenesis | -1,699 | **PROX1** | X |
| Cardiac atrium morphogenesis | -1,301 | **PROX1** | X |
| Pancreas development | -1,3979 | **PROX1** | X |
| Neural tube development | -1,301 | **PROX1** | X |
| Brain morphogenesis | -1,5229 | PAFAH1B1 |  |
| Embryo development ending in birth or egg hatching | -1,301 | FOXI1 |  |
| Regulation of transcription involved in cell fate commitment | -1,699 | **PROX1** | X |
| Positive regulation of metabolic process | -1,301 | ACACA |  |
| Myeloid dendritic cell activation | -1,3979 | HMGB1 |  |
| Venous blood vessel development | -1,699 | **PROX1** | X |
| Lymphatic endothelial cell differentiation | -1,699 | **PROX1** | X |
| Negative regulation of transcription from RNA polymerase II promoter | -1,5229 | EFNA1; HSBP1; ZNF281; **PROX1**; HMGB1 | X |
| Mitral valve development | -1,699 | EFNA1 |  |
| Positive regulation of muscle cell differentiation | -300 | MYOD1; BOC |  |
| Brain development | -2 | EPHA7; **PROX1**; PAFAH1B1 | X |
| Positive regulation of protein depolymerization | -1,3979 | KATNB1 |  |
| Positive regulation of macrophage derived foam cell differentiation | -1,301 | LPL |  |
| Positive regulation of blood vessel endothelial cell proliferation involved in sprouting angiogenesis | -1,699 | APLNR |  |
